# Treatment of Bifocal Cyst Hydatid Involvement in Right Femur with Teicoplanin Added Bone Cement and Albendazole

**DOI:** 10.1155/2015/824824

**Published:** 2015-07-06

**Authors:** Ozhan Pazarci, Zekeriya Oztemur, Okay Bulut

**Affiliations:** ^1^Reyhanlı State Hospital, 31000 Hatay, Turkey; ^2^Department of Orthopaedics and Traumatology, Cumhuriyet University School of Medicine, 58000 Sivas, Turkey

## Abstract

Although bone involvement associated with cyst hydatid is rarely seen, it can cause unintended results such as high recurrence rate, infection, sepsis, or amputation of relevant extremity. Because of this reason, its treatment is difficult and disputed. In the case of bifocal bone cyst hydatid in right femur, along with albendazole treatment, result of resecting cyst surgically and its treatment with teicoplanin with added bone cement is given. In conclusion, since the offered treatment method both supports bone in terms of mechanical aspect and also can prevent secondary infection, the method is thought to be a good and safe treatment approach.

## 1. Introduction

Hydatid disease due to* Echinococcus granulosus*, which is a condition also known as echinococcosis, usually manifests as cysts in the liver and lungs, but the larvae of this tapeworm can lodge anywhere in the body [1]. Skeletal involvement of* E. granulosus* is rare, occurring in only 0.28–3.1% of hydatidosis cases [1]. In a study, localisation of echinococci in the bone was reported in vertebra as 30%, in pelvis and hip as 20%, in femur and tibia as 15%, in humerus as 15%, and in phalanx as 5% [2]. There are difficulties in the diagnosis and treatment of cyst hydatid with bone involvement [3, 4].

In the reported case, combination of filling the cavity, which forms after the curettage of cyst and chlorhexidine washing, with antibiotic-loaded bone cement and albendazole treatment is discussed as a new approach for the treatment of bifocal cyst hydatid in femur.

## 2. Case

A 17-year-old female patient has a pain complaint in her hip and femur, which has increased especially during nights and by activity for almost three years and has also awoken the patient from sleeping. She had no known disease in her background; however, she had a story of keeping a dog in her house ten years ago. The patient was hitching during physical examination. Range of motion of right hip was full, and there was minimal pain in her hip movements. There were pain and sensitivity in right proximal and distal femur by pressure.

**Figure 1:**
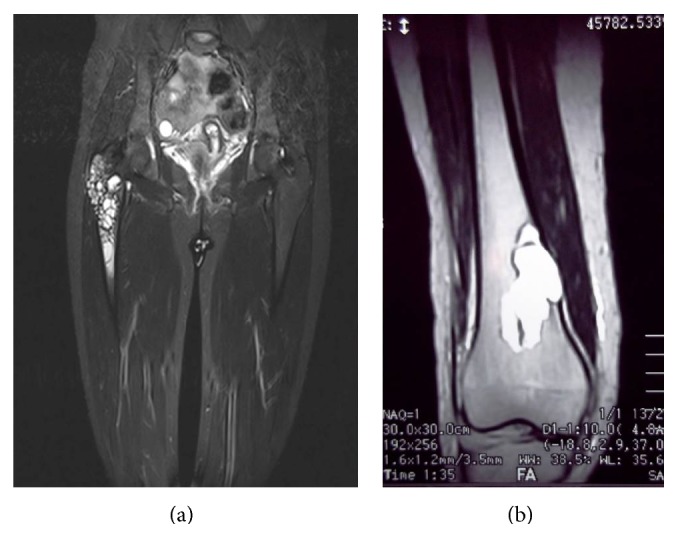
Preoperative MR images.

**Figure 2:**
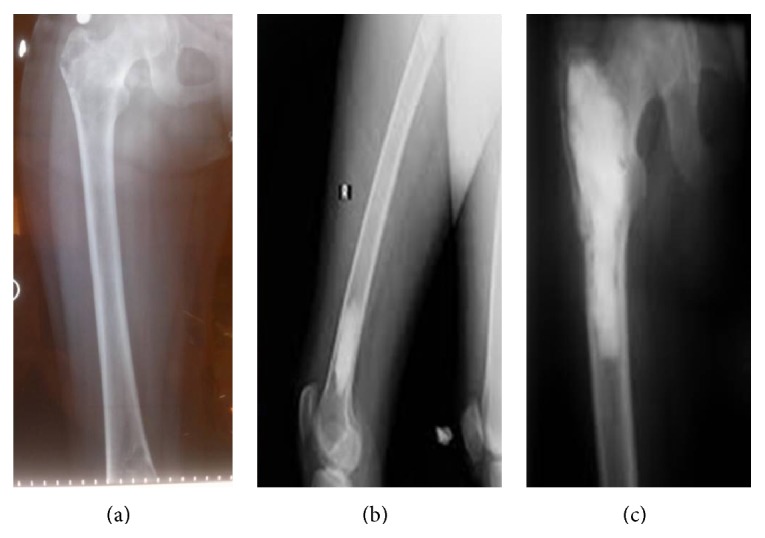
Preoperative and postoperative radiographs.

In the direct radiography, a cystic lesion with approximately 4 × 4 cm geographical borders displaying extension from right femur intertrochanteric area to subtrochanteric area was monitored (Figure 1). There was a cystic lesion which displays extension to the medial cortex with approximately 3 × 1 cm geographical borders in the right femur distal medial (Figure 1). In MRI, cystic mass lesion having multiloculated, trabecula, expansile characteristic and cystic lesion including septation with 6 × 2 cm lobule contour in right femur distal diaphysis along with apparent destruction in bone cortex are observed in the localization of right femur trochanter major, intertrochanteric area and femur 1/3 proximal diaphysis (Figure 2). Soft-tissue plans surrounding lesion were normal.

In the laboratory, sedimentation was 46, C-reactive protein was 1.28, white blood cell count was 7700, and indirect hemagglutination test was +(1/320). In the case, thought to have prediagnosis of cyst hydatid, abdominal and hepatobiliary ultrasonography, thorax computerised tomography, chest radiography, and vertebra radiography were evaluated as normal.

Proximal lesion was reached by opening approximately 2 × 6 cm cover from femoral cortex. After the curettage, forming cavity was washed with 4% chlorhexidine for 10 minutes and then filled with 40 gr bone cement with addition of 1200 mg teicoplanin in order to prevent the secondary infection. In the pathological examination, cuticula of cyst hydatid and its scolexes were observed and the treatment of albendazole 2 × 400 mg/day was started. Lesion in femur distal was cured after 14 days and was washed with 4% chlorhexidine and filled with bone cement. After that, albendazole treatment was completed in 28 days.

After the surgery, any problem regarding lesion area was not observed during follow-up; on the third month full weight was given to the patient, who was pressed by partial weight-bearing with crutches on the post-op second month. No complication was observed during the treatment except for minimal increase in liver enzymes and movement disability in the knee. After active/passive exercise program aimed at range of motion of knee joint, full range of motion was provided. During follow-ups made in the first and third year after treatment, no recurrence and serious complication were seen.

## 3. Discussion

The study conducted by Papanikolaou et al. [5] reported that bone involvement rate was between 0.2% and 4% and the great majority of them had vertebra involvement. In contrast to the case who has complaint of chronic hip pain, it is an insidious disease until complications such as paraplegia and pathological fracture develop [6]. As is in the reported case, the diagnosis is generally established intraoperatively. Preoperative radiological and serologic diagnosis methods are deprived of high sensitivity and specificity. Particularly in the cases with nonunion pathological fractures, having cystic lesions on any place of their bodies in endemic areas, diagnosis should be kept in mind [7].

Surgical treatment only may not be successful because of the difficulty of total excision and may increase the risk of dissemination. Recently treatment of osseous hydatid disease has been entirely surgical, and the aims are removal of the cyst and surrounding bone, replacement of bone defects with bone grafts or a prosthesis, avoidance of secondary infection, and prevention of recurrence [8–10]. Ideally, the lesions should be removed using the same technique in case of a malignant tumour [11, 12]. When the lesions are too extensive to allow complete excision, curettage and/or aspiration of a painful cyst are performed [11, 13], and chemotherapy effective against* E. granulosus* is given to prevent recurrences [11]. Clinical trials using benzimidazole compounds have shown promising but variable results. Both albendazole and mebendazole have demonstrated efficacy. A slightly greater efficacy related to rates of complete cure and improvement has been obtained with albendazole [14, 15]. We performed 28 days of treatment with albendazole. Different treatments have been described in the literature. Oxfendazole at 60 mg/kg and the combined therapies of oxfendazole  +  praziquantel (30 mg/Kg + 40 mg/Kg) and albendazole  +  praziquantel (30 mg/Kg + 40 mg/Kg) are successful agents that can be added to current control measures to interrupt the transmission of* Echinococcus granulosus*. It also demonstrates the potential of these antiparasitic drugs to be used in the treatment of cystic echinococcosis in a relatively short period of time, reducing the cost of the therapy [16]. They conclude that, in the future, combined therapy with praziquantel and albendazole could be successfully applied to humans as preoperative treatment, thereby avoiding accidental dissemination, due to the combination's protoscolicidal action [17].

In multiple bone involvements especially together with pelvis and hip involvement, infestation recurrence, sepsis, amputation, unsatisfactory results, and, also the worst of all, death complications are reported [18, 19]. For this reason, treatment planning and follow-up of multiple bone involvements are very important. In the reported case, there was bifocal cyst hydatid localisation in femur (Figure 2). When the treatment of the case is planned, it is considered that, especially for lesions on the same bone, there is a high possibility of development of pathological fracture during curettage or after the surgery. It was possible to provide a mechanical support by using an orthopaedic implant; however, it was planned to fill the cysts with bone cement after curettage procedure because of secondary infection risk.

It is postulated that the exothermic reaction of methylmethacrylate generates local hyperthermia which induces necrosis of surrounding tissue. The theory cannot exclude the possibility that the polymerisation of methylmethacrylate may produce a local chemical cytotoxic effect [20]. Cementation using methylmethacrylate has shown encouraging results in therapy of giant cell tumours of bone before [20]. Yildiz et al. [21] reported 15 cases of bone hydatidosis; ten of the patients had had surgery before in other clinics due to bone and/or soft-tissue lesions and suffered relapses which required further treatment. They performed curettage, swabbing with povidone iodine, and filling the defect with polymethylmethacrylate (PMMA) in 10 patients. Three of these had a recurrence after 5 years, but seven had no signs of relapse during a mean follow-up of 52 months. Different from that study, in our case, we tried to prevent infection and sepsis, which are the most serious complications, by providing highly antibiotic concentration around cyst as well as the mechanical support after curettage by adding teicoplanin to bone cement.

As a result, in infestations of cyst hydatid localised in the long bone, since filling the cyst with antibiotic-loaded bone cement after curettage of the cyst and washing it with chlorhexidine or betadine along with albendazole treatment can both support the bone mechanically and prevent the secondary infection, we consider the method as a good and safe treatment approach.
